# Antimicrobial susceptibility patterns of community-acquired uropathogenic *Escherichia coli*, Dublin 2010–2022

**DOI:** 10.1099/acmi.0.000633.v3

**Published:** 2023-08-24

**Authors:** Saied Ali, Laura Ryan

**Affiliations:** ^1^​ Department of Clinical Microbiology, St Vincent’s University Hospital, Elm Park, Dublin, Ireland

**Keywords:** antimicrobials, antimicrobial resistance, antimicrobial stewardship, community, *Escherichia coli*, surveillance, urinary tract infections

## Abstract

**Background.:**

*
Escherichia coli* is a common cause of urinary tract infections. Due to the increase in antimicrobial resistance (AMR) and global differences in antimicrobial susceptibility data, routine assessment of local antimicrobial susceptibility patterns is necessary to guide the selection of appropriate empirical therapy. The aim of this study was to evaluate the antimicrobial susceptibility patterns of community-acquired uropathogenic *Escherichia coli* within a catchment area in Dublin over a 13 year period, 2010–2022.

**Methods.:**

All mid-stream urine samples received from local general practitioners in which there was significant *E. coli* bacteriuria during the study period, 2010–2022, were included in the analysis. Antimicrobial susceptibility testing was performed by disc diffusion as per the European Committee on Antimicrobial Susceptibility Testing recommendations.

**Results.:**

An average of 11 407 urine samples per month had significant bacteriuria, with *E. coli* accounting for an average of 67 % of those. Overall AMR rates were highest for ampicillin (53.9 %), followed by trimethoprim (32.4 %), gentamicin (18.6 %), co-amoxiclav (16.5 %), ciprofloxacin (12.3 %), cephalexin (8.3 %), cefpodoxime (6.8 %) and nitrofurantoin (2 %). While rates appeared grossly static, statistically significant reduced resistance rates were noted for co-amoxiclav (r_s_=−0.95; *P*=<0.001), cephalexin prior to 2019 (r_s_=−0.783; *P*=0.013) and trimethoprim (r_s_=−0.639; *P*=0.019), with a statistically significant increase in non-susceptibility to cefpodoxime (r_s_=0.802; *P*=0.001).

**Conclusions.:**

In order to generate efficient empirical antimicrobial prescribing guidelines, knowledge of region-specific contemporaneous antimicrobial susceptibility patterns is pivotal. Our findings support the use of nitrofurantoin or cephalexin as empirical antimicrobial therapy within our setting.

## Data Summary

The authors confirm all supporting data, code and protocols have been provided within the article.

## Introduction

Urinary tract infections (UTIs) are among the most common bacterial infections worldwide, with uropathogenic *Escherichia coli* identified as the most common primary causative bacteria [1, 2].

Acute community-acquired (CA) uncomplicated cystitis or a lower UTI are typically a clinical diagnosis, characterized by suprapubic discomfort, dysuria and urinary frequency or urgency. Complicated or upper UTIs produce more systemic symptoms such as general unwellness, pyrexia, fatigue and flank tenderness [3].

Clinicians generally prescribe empirical antimicrobial therapy for CA infections; with urine microbial culture and antimicrobial susceptibility testing (AST) requested in cases of complicated or upper UTIs, recurrent UTIs, resistant infections or failure of empirical therapy. Furthermore, a pretreatment mid-stream urine (MSU) sample from all patients and from those having recently completed a course of antimicrobial therapy where the risk of antimicrobial resistance may be higher are endorsed by the Health Service Executive (HSE), Ireland, in addition to the aforementioned scenarios [4]. The rationale for empirical antimicrobial therapy is based on the assumed predictable antimicrobial susceptibility patterns of expected uropathogens [5]. As per the most recent guidelines from the HSE, published in September 2021, nitrofurantoin, cephalexin, trimethoprim and fosfomycin are the recommended empirical agents for uncomplicated UTIs and cephalexin, ciprofloxacin and co-amoxiclav for complicated UTIs in the Republic of Ireland [4].

However, the misuse of antimicrobials is a chief driver of acquired antimicrobial resistance (AMR) to regularly prescribed agents. AMR has been recognized by the World Health Organization (WHO) as an international emergency and one of the greatest potential threats to human health. Many first-line antimicrobials are now ineffective, with alternatives scare, more expensive and potentially more toxic [6]. It is now estimated that more than 35 000 people throughout the European Union/European Economic Area (EU/EEA) die from antimicrobial-resistant infections; a statistic now comparable to that of influenza, tuberculosis and human immunodeficiency virus infection and acquired immunodeficiency syndrome (HIV/AIDS) combined [7].

Due to the rise in AMR and global differences in antimicrobial susceptibility data, routine assessment of local antimicrobial susceptibility patterns is necessary to guide the selection of appropriate empirical therapy [8, 9].

The aim of this study was to evaluate antimicrobial susceptibility patterns of community-acquired uropathogenic *E. coli* within a catchment area in Dublin over a 13 year period.

## Methods

A retrospective analysis of the antimicrobial susceptibility patterns of community-acquired uropathogenic *E. coli* in a single Dublin teaching hospital over a 13 year period, 2010–2022 inclusive, was performed.

St Vincent’s University Hospital is a 600-bed academic teaching hospital providing care across over 50 different medical specialities in Dublin 4, Ireland.

All MSU samples received for microbiological analysis by general practitioners working within the catchment area were identified, as it was inferred that these samples represent CA-UTIs. Samples from which *E. coli* was isolated as the cause of significant bacteriuria were noted for analysis.

Significant bacteriuria was defined as culture of a single bacterial species, i.e. pure growth, from the urine sample at a concentration of 100 000 colony-forming units (c.f.u.) ml^−1^, associated with microscopic findings of at least 10 white blood cells per high power field, or a pure growth of 10 000 c.f.u. ml^−1^ when the white blood cell count was greater than 100 per high power field [10].

Microscopy is performed on all MSU samples to determine the presence and quantity of red and white blood cells. Specimens with a white blood cell count ≥10 are routinely cultured using a sterile 1 µl calibrated loop. Chromogenic CPSO (bioMérieux) agar plates are inoculated and incubated in air for 16–24 h at 35–37 °C. The majority of organisms are identified based on their appearance – colour and morphology. *E. coli* appears as burgundy or pink colonies [11]. Supplementary identification tests or use of the VITEK-MS matrix-assisted laser desorption/ionization time-of-flight (MALDI-TOF) are employed where necessary.

AST is performed using the disc diffusion method according to the European Committee on Antimicrobial Susceptibility Testing (EUCAST) recommendations [10]. Antimicrobials tested include ampicillin, co-amoxiclav, cephalexin, ciprofloxacin, nitrofurantoin, trimethoprim, gentamicin and cefpodoxime. Notably, in 2014, a larger disc diameter was established for co-amoxiclav AST for ‘uncomplicated’ UTIs. This was based on data that found that treatment success for ‘cystitis’, as opposed to systemic infection was likely even with relative resistance to co-amoxiclav. As such, AST results were determined using both the ‘systemic’ and ‘uncomplicated’ UTI breakpoints separately from 2014 onwards [12]. Additionally, AST for cephalexin was only performed for highly non-susceptible isolates until 2019, at which point all isolates were then routinely tested against cephalexin.

Antimicrobial susceptibility data were collected and processed through WHONET. IBM SPSS V.25 was used for further statistical analysis. Continuous variables were described as means and categorical variables as percentages and frequencies. Spearman’s correlation was performed to compare AMR trends. A *P*-value <0.05 was considered statistically significant.

## Results

**Fig. 1.**
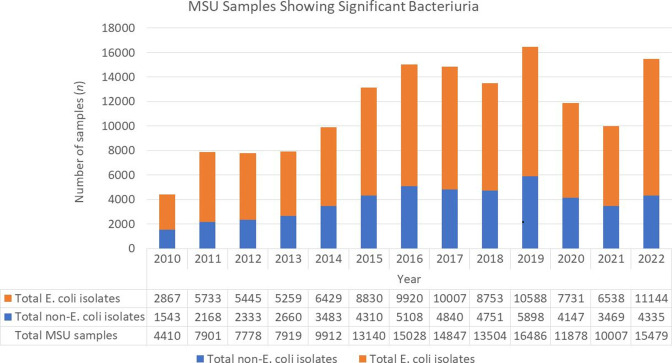
Proportion of uropathogenic *E. coli* isolated among all MSU processed, 2010–2022.

An average of 11 407 (IQR 7919–14 847) MSU samples per month were reported as significant bacteriuria over the study period, with *E. coli* accounting for an average of 67 % (IQR 65–67.4 %) of specimens. Proportions per month are depicted in Fig. 1.

Overall, the non-susceptibility rates, in descending order of isolated *E. coli *, were as follows: 58 % for cephalexin prior to 2019, 53.9 % for ampicillin, 32.4 % for trimethoprim, 18.6 % for gentamicin, 16.5 % for co-amoxiclav – uncomplicated, 16 % for co-amoxiclav – systemic, 12.3 % for ciprofloxacin, 8.3 % for cephalexin from 2019, 6.8 % for cefpodoxime and 2 % for nitrofurantoin.

**Fig. 2.**
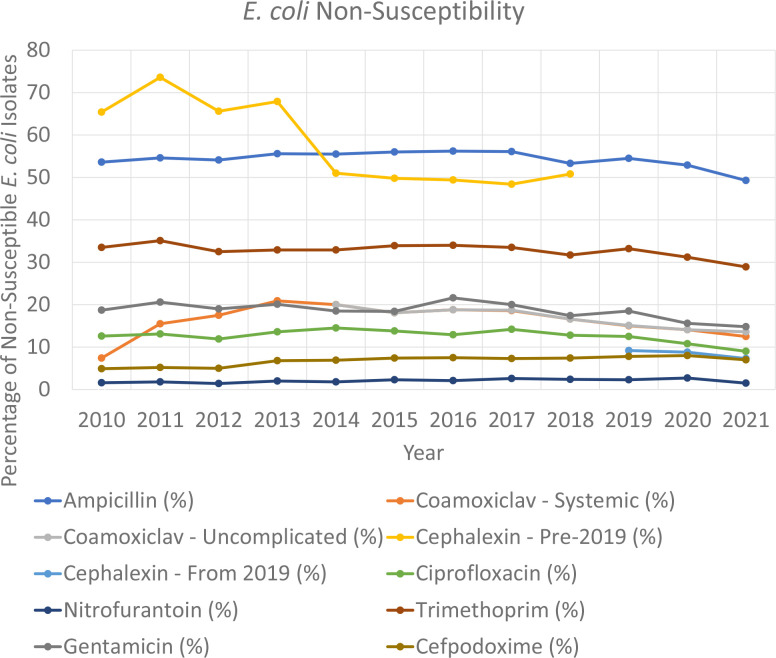
Non-susceptibility of uropathogenic *E. coli *, 2010–2022.

The evolution of antimicrobial susceptibility rates is shown in Fig. 2 and Table 1. While the rates appear generally static, with obvious differences related to testing practices as outlined in the Methods for, namely, cephalexin, statistically significant reduced rates of AMR were noted for co-amoxiclav – uncomplicated (r_s_=−0.95; *P*=<0.001), cephalexin prior to 2019 (r_s_=−0.783; *P*=0.013) and trimethoprim (r_s_=−0.639; *P*=0.019). However, a statistically significant increase in non-susceptibility to cefpodoxime (r_s_=0.802; *P*=0.001) was observed. All other trends were not statistically significant.

**Table 1.** Antimicrobial resistance rate of uropathogenic *E. coli* per year across tested antimicrobials

Antimicrobial	Antimicrobial resistance rate per year (%)												
	2010	2011	2012	2013	2014	2015	2016	2017	2018	2019	2020	2021	2022
Ampicillin	53.6	54.6	54.1	55.6	55.5	56	56.2	56.1	53.3	54.5	52.9	49.3	48.5
Co-amoxiclav – systemic	7.4	15.5	17.5	20.9	20	18.1	18.8	18.6	16.6	15	14.1	12.5	12.5
Co-amoxiclav – uncomplicated	nt	nt	nt	nt	20	18.1	18.8	18.7	16.6	15.1	14.1	13.6	13.2
Cephalexin	65.4	73.6	65.6	67.9	51	49.8	49.4	48.4	50.8	9.2	8.8	7.3	7.9
Ciprofloxacin	12.6	13.1	11.9	13.6	14.5	13.8	12.9	14.2	12.8	12.5	10.8	9	8.1
Nitrofurantoin	1.6	1.8	1.4	2	1.8	2.3	2.1	2.6	2.4	2.3	2.7	1.5	1.4
Trimethoprim	33.5	35.1	32.5	32.9	32.9	33.9	34	33.5	31.7	33.2	31.2	28.9	28.1
Gentamicin	18.7	20.6	19	20.1	18.5	18.4	21.6	20	17.4	18.5	15.6	14.8	18.8
Cefpodoxime	4.9	5.2	5	6.8	6.9	7.4	7.5	7.3	7.4	7.8	8	7	7.5

nt, not tested.

## Discussion

*E. coli* accounts for up to 90 % of CA-UTIs worldwide [2]. Empirical antimicrobial treatment is often initiated, as conventional AST requires a minimum of 48 h to facilitate required bacterial growth. However, this strategy of treatment may lead to the emergence of AMR due to inappropriate initial antimicrobial choices [9]. To control and mitigate the increasing prevalence of AMR, it is recommended that resistance rates should not exceed 20 % for proposed empirical agents [8, 13, 14].

Bacterial uropathogen epidemiology and resistance patterns show marked inter-regional variability, and rates of AMR are continually changing due to diverse antimicrobial treatment regimens. *In vitro* antimicrobial susceptibility data for uropathogenic *E. coli* from Spain and Portugal have reported higher AMR rates than other European countries, and similarly the USA when compared to Canada [9]. Even in the same country, the antimicrobial susceptibility patterns of isolates are discrepant. For that reason, and in order to generate efficient empirical antimicrobial prescribing guidelines, knowledge of region-specific contemporaneous antimicrobial susceptibility patterns is pivotal [15, 16].

The North American Urinary Tract Infection Collaborative Alliance (NAUTICA) study evaluated antimicrobial susceptibility data for outpatient *E. coli* urinary isolates obtained from various regions in the USA and Canada. AMR rates to trimethoprim, nitrofurantoin and ciprofloxacin were found to be 21.3, 1.1 and 5.5%, respectively [17].

The later ECO.SENS study conducted in Europe and Canada reported *E. coli* AMR rates of 30 % for ampicillin, 15 % for trimethoprim and <3 % for co-amoxiclav, nitrofurantoin, gentamicin and ciprofloxacin [18].

Our findings report higher AMR rates across all antimicrobials compared to the abovementioned, likely due to differing prescribing habits, antimicrobial stewardship (AMS) interventions and local and national policy [15–18].

Looking at the most recent report from the European Antimicrobial Resistance Surveillance Network (EARS-Net) for invasive *E. coli* isolates studied in 2021, aminopenicillin – including ampicillin – AMR was reported at 53.1 %, fluoroquinolone – including ciprofloxacin – at 21.9 % and aminoglycoside – including gentamicin – at 9.6 %; all of which have been decreasing since 2017 [19]. Our findings show a similar picture for ampicillin, with an observed AMR rate of 53.9 %, but a lower AMR rate for ciprofloxacin (12.3 %) and a higher AMR rate for gentamicin (18.6 %).

An 11 year retrospective study conducted in Dublin 1999–2009 identified ampicillin and trimethoprim as unsuitable empirical choices for CA-UTIs with a mean AMR rate of 62.1 and 33.5% respectively. Nitrofurantoin was the most active agent, with an AMR rate at 2.7 %, while ciprofloxacin and co-amoxiclav remained reasonable options, but a steady increase in AMR rates over the time period was noted [8]. Similar findings were published in another review of *E. coli* antimicrobial resistance patterns from a different Dublin catchment area from 2005 to 2014 [20].

While these reviews were published from different institutions serving different areas during a different time period, our findings are very similar. Ampicillin and trimethoprim remain unsuitable empirical therapeutic options, with AMR rates of 53.9 and 32.4% respectively. Interestingly, non-susceptibility to trimethoprim decreased steadily over the study time period, at a rate found to be statistically significant. This is likely due to reduction in prescriptions based on knowledge of a high level of circulating trimethoprim AMR. On the other hand, nitrofurantoin continues to boast the lowest AMR rate of 2 %.

AMR to fluoroquinolones and co-amoxiclav remains of global concern, with worldwide rates as high as 60 and 70%, respectively [14, 19, 21]. Encouragingly, ciprofloxacin and co-amoxiclav remain viable options, with AMR rates of <20 % and the previously noted climb in AMR rates was not evident in this contemporaneous study. This may be due in part to the removal of both co-amoxiclav and ciprofloxacin from HSE prescribing guidelines for uncomplicated UTIs, as well as the Green Red Antibiotic Quality Improvement Initiative for Community Prescribers. This movement sought to encourage general practitioners to prescribe ‘green’ antimicrobials, which were deemed to be effective, narrow-spectrum agents with fewer side effects and less likely to promote the development of AMR, compared with ‘red’ agents. Co-amoxiclav and ciprofloxacin were categorized as ‘red’ agents, with nitrofurantoin, trimethoprim, amoxicillin and cephalexin being ‘green’ agents. The regular feedback to general practitioners on the quality of their antimicrobial prescriptions was highly motivating, with a decrease of as much as 28 % in red prescriptions since its inception in 2020 [22]. Additionally, it is reassuring with the introduction of clinical breakpoints for co-amoxiclav for ‘uncomplicated’ UTIs, that a statistically significant decrease in non-susceptibility was observed for this antimicrobial for this condition.

Published AMR surveillance data for cephalexin for uropathogenic *E. coli* are scarce. The initial high AMR rates displayed in Fig. 2 are directly related to the fact that only multidrug-resistant organisms were tested against cephalexin at that time; so understandably there is selection bias among that set of isolates. Despite this, the overall rate of AMR decreased over time, and persisted at a statistically significant level until cephalexin AST became routine for all uropathogens in 2019; and with an average AMR rate of 8.3 % from 2019, cephalexin is a reasonable empirical antimicrobial choice for CA-UTIs.

The AMR rate of 18.6 % for gentamicin is consistent with previously published studies reporting non-susceptibility rates of 0.3–36.6 % [18, 19], with resistance being attributed to the inappropriate administration of a single dose for the outpatient treatment of CA-UTIs [13].

Cefpodoxime, although not a therapeutic antimicrobial, serves as a marker for β-lactamase production, specifically extended-spectrum β-lactamases (ESBLs), which confer resistance to β-lactam classes of antimicrobials. Non-susceptibility to cefpodoxime results in the isolate being tested for ESBL production using VITEK2 (bioMérieux), which simultaneously assesses of the inhibitory effects of cefepime, cefotaxime and ceftazidime, alone and in the presence of clavulanate, the latter of which inhibits ESBL production [23].

Unfortunately, data regarding the exact proportion of ESBL isolates during this study period are unavailable due to electronic system errors. Nonetheless, a relatively benign overall AMR rate of 6.8 % for cefpodoxime over the 11 years may not be of concern, but the statistically significant increase in this rate should not be overlooked, as it may suggest an increasing proportion of ESBL isolates, usually driven by broad-spectrum antimicrobial use, and emphasizes the importance of AMS.

In Ireland, most uncomplicated CA-UTIs are managed by primary care with short courses of empirical antimicrobials. In many cases, urine samples are only sent for microbiological evaluation following treatment failure and recurrent or relapsing infection, and therefore the levels of AMR observed amongst community isolates may overestimate the true rate of AMR in the community.

As our study reports on antimicrobial susceptibility data from a single institution, the results are not readily generalizable. Furthermore, as the focus was on CA-UTIs, assessment of healthcare-associated UTIs represents an area of future investigation. Similarly, we did not correlate AMR rates with antimicrobial consumption data, direct community AMS interventions, infection prevention and control initiatives or AMR in animal and environmental reservoirs as part of One Health.

Surveillance of local *E. coli* AMR data will not only highlight emerging trends in resistance, but inform empirical antimicrobial prescribing guidelines, as well as recommendations for antimicrobial prophylaxis in urological procedures. Furthermore, it enables clinicians to make informed decisions regarding antimicrobial therapy for all infections likely to be caused by this organism.

## Conclusion

AMR among uropathogens is increasing worldwide. Knowledge of antimicrobial susceptibility data in specific geographical locations remains an important factor in determining appropriate evidence-based empirical antimicrobial treatment. Our findings support the use of either nitrofurantoin or cephalexin as appropriate empirical antimicrobials for the treatment of CA-UTIs, as well as the need to continue rigorous AMS initiatives in the fight against AMR. The statistically significant increase in AMR to cefpodoxime warrants ongoing surveillance.
